# The Biological Response of Spermidine Induced by Ionization Radiation

**DOI:** 10.3390/molecules17010145

**Published:** 2011-12-23

**Authors:** Changhyun Roh, Dong-Kyung Yu, Insub Kim, Sung Kee Jo

**Affiliations:** Radiation Research Division for Biotechnology, Advanced Radiation Technology Institute (ARTI), Korea Atomic Energy Research Institute (KAERI), 1266, Sinjeong-dong, Jeongeup, Jeonbuk 580-185, Korea; Email: dkyoo@kaeri.re.kr (D.-K.Y.); Insub@kaeri.re.kr (I.K.); skjo@kaeri.re.kr (S.K.J.)

**Keywords:** ionizing radiation, metabolomics, polyamines, biological response

## Abstract

Globally, there are concerns over the risks associated with radiation exposure, so it is important to understand the biological effects of radiation exposure. Driven by the need to detect the presence of radiation exposure, biomarkers to monitor potential exposure after radiological accidents can be developed and would be extremely valuable for biological response. In this study, the behavior of spermidine as a biomarker was investigated in a C57BL/6 mouse model exposed to an acute whole-body sublethal dose of 6 Gy. The spermidine content values in serum increased for up to two days after 6 Gy irradiation. However, the enhanced spermidine content observed on day +3 in irradiated mice returned to normal levels on the subsequent five days. The result indicates that spermidine can be used as a biomarker of biological response to radiation exposure.

## 1. Introduction

Ionizing radiation (IR) has inevitably become a major public health concern due to potential exposure from artificial sources such as radiological medical usage and from natural sources like space travel [1,2]. Especially, radiation exposures from nuclear and radiation accidents and the threat of terrorism using radioactive isotopes are big issues [2,3]. The polyamines putrescine, spermidine, and spermine play a pivotal role in living organisms [4,5,6]. Polyamines are low molecular weight organic polycations displaying important biological activities and mediating a multitude of *in vivo* processes. In general, the polycationic nature of polyamines is important for their biological activities and their *in vivo* interaction with multiple kinds of molecules such as polyanionic nucleic acids and proteins.

Regarding their functions, polyamines play a critical role in cell metabolism [7], cell proliferation [8], and cell differentiation [9] through cellular processes including the metabolic pathways of synthesis, degradation and transport [10,11]. Especially, the polyamine spermidine is an aliphatic polycation present in all cells, where it has pleiotropic effects that allow its linkage to DNA, RNA, and proteins. Spermidine plays a regulatory role in a number of steps of cell metabolism in living organisms.

Understanding of cellular responses to ionizing radiation is essential for the development of predictive biomarkers useful for assessing radiation exposure in animals. Usually, radiation exposure at 6 Gy dose leads to the death of the irradiated animal due to acute radiation injury. In this study, we elucidated the behavior of spermidine in a C57BL/6 mouse model exposed to an acute whole-body sublethal dose of 6Gy. To the best of our knowledge, this is first report suggesting that spermidine can be used as a good biomarker of biological response to ionizing radiation lethality.

## 2. Results and Discussion

**Figure 1:**
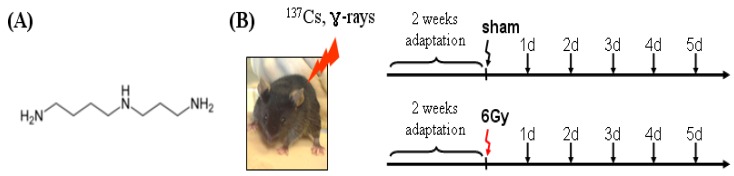
The chemical structure of spermidine(**A**) and experimental design of the mouse (C57BL/6) model response to ionizing radiation (**B**).

Eight-week-old mice were placed in self-made containers and exposed to whole-body γ-irradiation from a ^137^Cs source (Gammacell 40). Acutely irradiated mice were exposed to 6 Gy of radiation (1.1 Gy/min). Every day from the beginning of the exposure, mice were sacrificed and the serum was extracted from the abdominal vein. At this time, day-matched normal mice were used as sham group. A schematic outline of the gamma irradiation procedure in mice is summarized in Figure 1.

**Figure 2:**
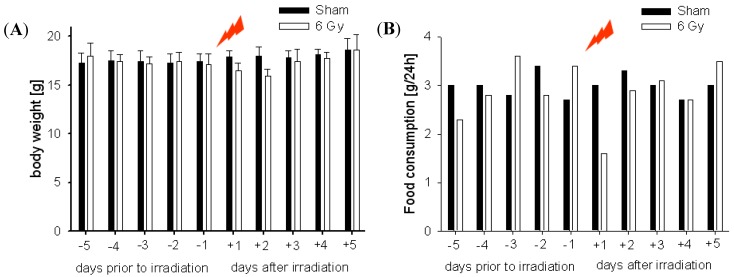
Trends in body weight on days -5 and +5 (**A**) in food consumption (**B**). Filled bars and open bars are for sham-irradiated and irradiated C57BL/6, respectively.

There was a marked reduction in body weight in the irradiated mice on days +1 and +2. The body weight of the control mice (sham group) increased during the study. In contrast, the irradiated mice experienced a 7.02% loss in body weight from 17.1 g to 15.9 g. However, body weight was recovered to normal levels on the subsequent +3 day. We monitored the food intake on a daily basis for 5 days. During the monitoring period, the food intakes of mice were significantly decreased for 1 day after 6 Gy irradiation. There was a trend toward lower food consumption in the 6 Gy irradiated mice post-irradiation, however, the enhanced food consumption observed on day +3 in irradiated mice returned to normal levels on the subsequent days. For food intake, there were noticeable differences in normal and gamma irradiation groups at +1 day (Figure 2B).

**Figure 3:**
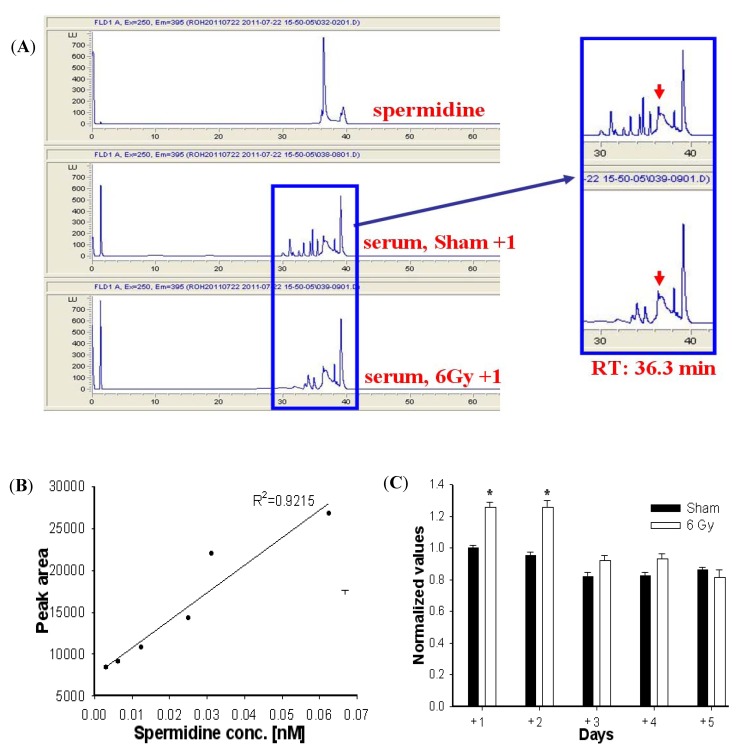
HPLC analysis for spermidine change (**A**) Spermidine standard curve (**B**) Spermidine concentration in the serum of sham and irradiated C57BL/6 model (**C**).

High performance lipid chromatography (HPLC) was performed to analyze the spermidine content. Figure 3(A) shows the spermidine profile of authentic and serum samples from sham and gamma-irradiated mice. The retention time (RT) of spermidine as a biomarker was 36.3 min. The standard curve of authentic spermidine is presented in Figure 3(B). The spermidine content in serum increased for up to 2 days after 6 Gy irradiation, but the enhanced spermidine content observed on day +3 in irradiated mice returned to normal levels during the subsequent 5 days. In Figure 3(C), the mice irradiated at different days showed the change of spermidine content. The normalized values represented the quantitative comparison of the sham and gamma-irradiated samples in spermidine content (* *p* < 0.05).

## 3. Experimental

### 3.1. Mice

C57BL/6 female mice were used in this study. Young mice (6 weeks of age) were purchased from Orient Bio Co., Ltd. (Charles River Technology, Songham, Republic of Korea). The mice (3–4 per cage) were kept under specific pathogen-free conditions at 23 ± 3 °C and 50 ± 10% relative humidity, and were provided with nutritional chow 5L79 (PMI Nutrition LLC, St Louis, MO, USA). The mice were given free access to water and were fed on a standard diet. All procedures were conducted in accordance with the ethical guidelines in the Animal Care Act, prepared by the Ministry of Agriculture and Forestry, Republic of Korea.

### 3.2. Ionizing Radiation

The mice were exposed whole-body to ^137^Cs γ-rays without physical restraint or anesthesia in a plastic chamber using a Gamma Cell 40 Exactor (Nordion International Inc., Ottawa, ON, Canada). Groups of two-month-old female C57BL/6 mice were exposed whole-body to ^137^Cs γ-rays as an acute (6 Gy) dose. After exposure to γ-irradiation, the groups of mice were sorted into individual cages and monitored daily for the development of symptoms of radiation sickness and mortality. The mice were divided into ten groups of four mice in each group. Two month-old female C57BL/6 mice were exposed to ^137^Cs γ-rays at acute 6 Gy (1.1 Gy/min) dose. And, the mice were monitored daily for the development of symptoms of radiation sickness and mortality. Sham irradiation was performed by placing C57BL/6 mice similarly into the irradiator but without exposure to the gamma-ray source.

### 3.3. Spermidine Analysis by HPLC

HPLC analysis of spermidine was performed using precolumn derivatization of the amine groups with the AccQ-Fluor^TM^ reagent kit (Waters Corp., Milford, MA, USA) following the manufacturer’s instructions. In each case, serum (10 μL) was included in the derivatization reaction. Reactions were performed in Accu-Fluor^TM^ borate buffer in a total volume of 100 μL and heated to 55 °C for 10 min. The derivatized spermidine samples was analyzed by HPLC using a reverse-phase modified C18 column (Waters Corp., Milford, MA, USA) on a Agilent HPLC System. The system was operated at 37 °C and equilibrated with Eluent A (70 mM acetic acid, 25 mM triethylamine, pH 4.82) at 1 mL/min. Elution was performed using the following linear gradients of Eluent B (80% acetonitrile): 7% for 15 min, 19% for 3 min, 33% for 15 min, finally 100% for 65 min. Spermidine was monitored by fluorescence (FLD detector) with a 250 nm excitation filter and a 395 nm emission filter and identified by comparison of retention times with a known standard that was derivatized and analyzed in parallel with the serum. Precise quantification of spermidine was performed using the internal standard and a calibration curve of spermidine (7–70 nM).

### 3.4. Statistical Analysis

Statistical analysis was done using a one way analysis of variance (ANOVA) and inter group comparisons were made using Tukey's multiple comparison test. Difference with a *p* value of less than 0.05 was considered as significant.

## 4. Conclusions

The polyamine spermidine has been linked to cell metabolism in living organisms. Little is known about spermidine changes in response to sublethal doses of ionizing radiation. The results suggest that the spermidine appears to be a good biomarker to estimate radiation damage lethality.
